# Copolymeric Hydrogel-Based Immobilization of Yeast Cells for Continuous Biotransformation of Fumaric Acid in a Microreactor

**DOI:** 10.3390/mi10120867

**Published:** 2019-12-10

**Authors:** Tadej Menegatti, Polona Žnidaršič-Plazl

**Affiliations:** 1 Faculty of Chemistry and Chemical Technology, University of Ljubljana, Večna pot 113, SI-1000 Ljubljana, Slovenia; tadej.menegatti@fkkt.uni-lj.si; 2 Chair of Microprocess Engineering and Technology—COMPETE, University of Ljubljana, Večna pot 113, SI-1000 Ljubljana, Slovenia

**Keywords:** immobilization, whole-cell biocatalysis, microbioreactor, copolymeric hydrogel, *Saccharomyces cerevisiae*

## Abstract

Although enzymatic microbioreactors have recently gained lots of attention, reports on the use of whole cells as biocatalysts in microreactors have been rather modest. In this work, an efficient microreactor with permeabilized *Saccharomyces cerevisiae* cells was developed and used for continuous biotransformation of fumaric into industrially relevant L-malic acid. The immobilization of yeast cells was achieved by entrapment in a porous structure of various hydrogels. Copolymers based on different ratios of sodium alginate (SA) and polyvinyl alcohol (PVA) were used for hydrogel formation, while calcium chloride and boric or phenylboronic acid were tested as crosslinking agents for SA and PVA, respectively. The influence of hydrogel composition on physico-chemical properties of hydrogels prepared in the form of thin films was evaluated. Immobilization of permeabilized *S. cerevisiae* cells in the selected copolymeric hydrogel resulted in up to 72% retained fumarase activity. The continuous biotransformation process using two layers of hydrogels integrated into a two-plate microreactor revealed high space time yield of 2.86 g/(L·h) while no activity loss was recorded during 7 days of continuous operation.

## 1. Introduction

Microreactor technology has recently been attracting increasing attention due to many advantages such as better heat and mass transfer, as well as more efficient mixing and overall better process control [1,2]. The integration of biocatalysts within microstructured devices opens the opportunity for the efficient valorization of biomass and sustainable transformation of chemical compounds [3]. Therefore, it is expected that the implementation of micro(bio)reactor technology in chemical and pharmaceutical industry will increase in the near future [4,5,6,7].

Immobilization of enzymes or whole cells has been an essential part of many biocatalytic process developments since it enables the reusability of the often-expensive biocatalyst and, thereby, prolongs its lifetime. Advantages of the immobilized biocatalyst compared with the free one also includes protection from harsh environmental conditions such as pH, temperature, organic solvent, and toxic compounds, as well as relative ease of product separation [8,9]. The selection of a suitable matrix for cell immobilization is essential for successful application of the immobilized cells for the bioprocess. Various techniques have been developed for biocatalyst immobilization, including adsorption or covalent linking to insoluble materials, entrapment in polymeric hydrogels and encapsulation in membranes [10,11,12,13].

Hydrogels are polymeric networks having high affinity for water but are prevented from dissolving due to their chemically or physically cross-linked structure [14]. Among polymers used for hydrogels applied in biocatalyst immobilization there are biopolymers such as alginate, chitosan, κ-carrageenan and cellulose, or synthetic ones such as polyvinyl alcohol (PVA) and polyacrylamide (PAM) [15,16,17,18,19,20]. Each polymer has its benefits, but also drawbacks, such as poor mechanical strength and durability, toxicity to microorganisms, or high cost [21]. Immobilization of microorganisms in spherical polymeric matrices is a promising tool for improvement of bioprocess efficiency. Among the most promising hydrogels that have already been applied in microreactor technology are lens-shaped PVA particles [12]. There are also many reports on enzyme entrapment inside porous structures of alginate and PVA beads, but very few have tried to combine the polymers to form a hydrogel with better overall properties [22]. Alginate with the crosslinking agent CaCl_2_ has been commonly used for biocatalyst immobilization due to its high biocompatibility and simple gelation [15,23,24]. However, calcium alginate is not stable and has poor mechanical properties. The addition of PVA, which is a synthetic polymer that is non-toxic, cheap and demonstrates strong mechanical properties, results in a copolymer that has better thermal, mechanical and chemical stability [25].

Whole cells offer distinct advantages over the isolated enzymes, particularly if complex transformations involving cofactors are considered. They are also less susceptible to denaturation and are significantly cheaper than isolated enzymes [26]. However, using a whole-cell biocatalyst also has drawbacks in higher mass transfer limitations of the substrate and the product through the cell membrane. To overcome mass transfer limitations, the cell membrane can be permeabilized. Permeabilization of the cells enables faster diffusion of the substrate or product across the cell membrane [27].

In this work, biotransformation of fumaric to L-malic acid by permeabilized *Saccharomyces cerevisiae* cells was used as a model reaction. L-malic acid has many applications in food, beverage, pharmaceutical and chemical industries, as well as in medicine [28]. The most widespread industrial production of L-malic acid is chemical synthesis from maleic or fumaric acid at high temperature and high pressure that yields a DL- racemic mixture [29]. On the other hand, enantiomerically pure L-malic acid is produced by enantioselective hydration with fumarase using either cells or isolated enzymes in a free or immobilized form [6,30,31,32].

The aim of this study was to develop a microreactor with whole-cell biocatalyst immobilized in a copolymer hydrogel matrix. Optimization of the hydrogel as an immobilization carrier was investigated by studying the effect of PVA concentration and various crosslinkers on physico-chemical properties of hydrogels. Furthermore, thin hydrogel films with yeast cells were integrated in a two-plate microreactor and used for L-malic acid production. Optimization of the biotransformation process in terms of hydrogel film loading and temperature was performed. Finally, system stability was studied during several days of continuous biotransformation.

## 2. Materials and Methods

### 2.1. Chemicals

Fumaric acid, hexadecyltrimethylammonium bromide (CTAB), 4-(2-hydroxyethyl)-1-piperazine-ethanesulfonic acid (HEPES), sodium alginate (SA) and phenylboronic acid (PBA) were all from Sigma Aldrich (St. Louis, MO, USA). Polyvinyl alcohol (PVA, MW = 13,000–23,000) was purchased from Acros organics (Morris Plains, NJ, USA). CaCl_2_ was from Carlo Erba reagents (Milan, Italy) and boric acid (BA) was from Kemika (Zagreb, Croatia). Demineralized water was used in all experiments.

### 2.2. Microorganism Cultivation and Preparation

*S. cerevisiae* MZKI K86, obtained from the culture collection of the National Institute of Chemistry (Ljubljana, Slovenia), was cultured in a medium containing 10 g/L yeast extract, 20 g/L peptone, and 20 g/L saccharose (pH 5.5) in 250 mL Erlenmeyer flasks. Cells were grown overnight at 30 °C and 150 rpm in a rotary shaker [31]. After harvesting by centrifugation, cells were washed thrice with water, permeabilized with 0.2% CTAB for 6 min and then again washed twice with water. Cells were then resuspended in 0.1 M HEPES buffer (pH 7) in a final concentration of about 10^9^ cells/mL.

### 2.3. Cell Immobilization in Sodium Alginate-Polyvinyl Alcohol (SA-PVA) Copolymeric Hydrogel

For copolymeric-hydrogel preparation, the aqueous suspension of SA and PVA was mixed and heated to 60 °C in order to completely dissolve both polymers and then cooled to 35 °C. In the case of biocatalyst immobilization, the suspension of permeabilized *S. cerevisiae* cells was added at this stage to yield final cell concentration of 1.64 × 10^8^ cells/mL, followed by a thorough mixing. Separately, CaCl_2_ and boric/phenylboronic acid were mixed in demineralized water to obtain the crosslinking solution. A volume of 1364 mL of the copolymer solution was then poured in a small Petri dish and the crosslinking agent was poured on top of it to start the crosslinking process. After 60 min, the layer of formed hydrogel was washed with demineralized water prior to use.

Hydrogel characterization was performed for different compositions of copolymers combining 2% (*w*/*v*) of SA and PVA in concentrations from 4–12% (*w*/*v*) as shown in Table 1. Besides, two crosslinking solutions were tested; one consisting of 2% (*w*/*v*) CaCl_2_ and 0.5% (*w*/*v*) BA, and the second one consisting of 2% (*w*/*v*) CaCl_2_ and 2% (*w*/*v*) PBA, which is reflected from the hydrogel denomination evident in Table 1.

### 2.4. Characterization of SA-PVA Copolymeric Hydrogel

The effect of PVA concentration and crosslinking reagents on swelling and rheological properties of the hydrogels was determined. Hydrogels were first carefully dried by wiping off the surface liquid using a tissue, after which the sample was weighed. Samples were then immersed in 0.5 L of water and at specific times they were wiped in the same manner as before and weighed. Equation (1) was used to determine the swelling:

(1) $$\mathrm{Swelling}\;\mathrm{ratio}=\frac{W_{t}-W_{0}}{W_{t}}\cdot 100\%$$

where *W*_t_ presents sample weight after the certain incubation time in water, while *W*_0_ states for the sample weight before the immersion in the water.

The hydrogel rheology was measured with an Anton Paar Physica MCR302 rheometer in a parallel plate configuration. Each sample was analyzed with a frequency sweep from 0.1 to 100 s^−1^. All samples were prepared in 1 mm thickness and with 40 mm diameter. As a measure of the ratio of energy lost to energy stored during cyclic deformation, the loss tangent (tan δ) was calculated:

(2) $$\tan\;\delta =\frac{G^{\prime \prime }\left(loss\;modulus\right)}{G^{\prime }\left(storage\;modulus\right)}$$

where the loss modulus (*G*″) relates to the energy dissipated from the sample as heat when shear is applied, representing the viscous characteristics of the sample. The storage modulus (*G*′) relates to the energy elastically stored in the sample when shear is applied, representing the elastic characteristics of the sample [33].

### 2.5. Microreactor Assembly

**Figure 1:**
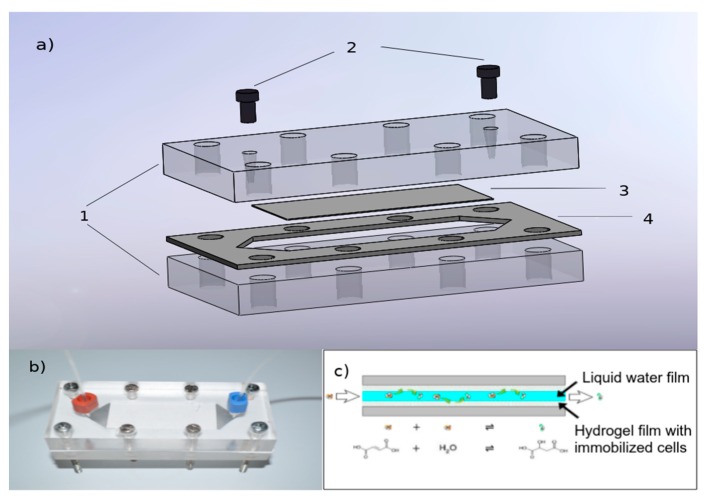
(**a**) Schematic presentation of a microbioreactor assembly with the main components: 1—poly(methyl methacrylate) (PMMA) plates; 2—high pressure tube fittings; 3—copolymer hydrogel layer with immobilized yeast cells; 4—non-compressible polytetrafluoroethylene (PTFE) spacer, (**b**) a picture of a microbioreactor between two plates with inlet and outlet fittings and (**c**) a schematic presentation of fumaric to L-malic acid biotransformation in the microbioreactor.

Microreactor (schematic presentation in Figure 1a and realistic picture in Figure 1b) was assembled using two poly(methyl methacrylate) (PMMA) plates where the upper one had inlet and outlet holes connected to perfluoroalkoxy (PFA) tubes (1.59 mm OD; 0.5 mm ID) via high-pressure polyetheretherketone (PEEK) tube fittings (Vici AG International, Schenkon, Switzerland). A channel for the single-layer microreactor was carved out of 0.5 mm non-compressible polytetrafluoroethylene (PTFE) film (DASTAFLON^®^ 1620, Dastaflon, Medvode, Slovenia) using a scalpel [12], while for a double-layered hydrogel-based microreactor, 0.7 mm PTFE film was used in order to obtain the same working volume and channel depth of 300 μm. A 5 cm long and 2 cm wide hydrogel layer covered the rectangular part of the hexagon-shaped channel carved from the PTFE film. The microreactor channel, therefore, consisted of the hydrogel filling the rectangular part between two empty triangular chambers, which provided unobstructed inlet and outlet of the fluid through the microreactor. One or two layers of various hydrogels with immobilized yeast cells were inserted in the rectangular part of the channels and glued on inner walls of the PMMA plates with 45 μm thick polypropylene double-sided adhesive tape donated by Adhesives Research (Glen Rock, PA, USA) in a way that inlet solution would flow between both hydrogel layers or between the single hydrogel layer and the PMMA plate through the reactor. Each hydrogel layer was about 200 μm thick. In the case of a single or double hydrogel layer, a microbioreactor had a working volume of 350 (±5) μL, as assured by the PTFE gasket. Each hydrogel layer contained 164 (±1.3) mg of wet yeast cells which corresponds to 33 (±0.8) mg of dry yeast cells with specific enzyme activity of 15.66 U/g, yielding final biocatalyst concentration of 94.25 (±3.7) and 188.5 (±4.5) mg_dw of cells_/mL of reactor volume in the case of a single or double-layered hydrogel-based microbioreactor, respectively.

### 2.6. Determination of the Immobilization Effectiveness Factor

In order to determine the immobilization effectiveness factor, the activity of free and immobilized biocatalyst had to be measured. For free biocatalyst activity determination, a batch bioprocess in a flask with 10 mL of 5 mM fumaric acid in 0.1 M HEPES buffer with 15 mg/mL concentration of free permeabilized yeast cells was performed. Activity was measured after 5 min which was within the period of the initial (constant) reaction rate. For the estimation of the immobilized biocatalyst activity, the process parameters stayed the same except that the same concentration of permeabilized yeast cells has been entrapped in a 2 mL of the hydrogel in the form of the thin layer prior to reaction. The batch biotransformations were performed at 30 °C with at 100 min^−1^ shaking. Immobilization effectiveness factor, *η*, was calculated using Equation (3):

(3) $$\eta =\frac{Immobilized\;enzyme\;activity}{Free\;enzyme\;activity}$$

### 2.7. Biotransformation in a Microreactor

A microbioreactor with immobilized permeabilized *S. cerevisiae* cells was prepared by implementation of a porous layer of the hydrogel with cells between two plates as shown in Figure 1c, or with a single hydrogel layer on the bottom as described in Section 2.5. The reaction was performed by pumping 5 mM solution of fumaric acid in 0,1 M HEPES buffer (pH 7) through the microreactor between the hydrogel. The reactions were carried out at different temperatures ranging from 22 to 60 °C, while flow rates ranged from 2 to 20 μL/min. Flow was controlled by the high-pressure syringe pumps (PHD 4400 Syringe Pump Series) from Harvard Apparatus (Holliston, MA, USA). After reaching steady-state conditions, outflows from microreactors were collected and analyzed as specified below. Conversions were calculated based on the inflow and outflow substrate concentrations. The volumetric productivities were calculated from analyzed fumaric acid concentrations assuming the formation of an equimolar amount of product and considering the retention time within the microbioreactor [6]. The biocatalyst productivities were calculated from the outlet fumaric acid concentration and cell dry weight within the given microreactor volume [6]. The percentage of active yeast cells inside the hydrogel layers was calculated from the biocatalyst specific activity obtained in a batch process using free permeabilized *S. cerevisiae* cells, and the biocatalyst productivity of the cells immobilized within the microbioreactor.

### 2.8. The Effect of Temperature on Biotransformation in a Microreactor

Biotransformation of fumaric to L-malic acid was performed with immobilized permeabilized cells in a hydrogel consisting of 8% (*w*/*v*) of PVA and 2% (*w*/*v*) of SA, crosslinked with PBA for 60 min. Several runs of biotransformation were accomplished by pumping 5 mM fumaric acid dissolved in 0.1 M HEPES at the flow rate of 12 μL/min. A microbioreactor was set in a thermostatic water bath at temperatures between 22 and 60 °C and the samples were taken at the outlet after reaching a steady state at least three times at each tested conditions.

### 2.9. Determination of Biotransformation System Stability

Operational stability of the microreactor system was assessed by performing the biotransformation continuously for several days at room temperature. The flow of 5 mM fumaric acid aqueous solution was set at 5 μL/min and samples were taken at 24 h intervals; the conversion of fumaric acid was calculated from substrate concentrations analyzed at the outlet of the microreactor and the inlet concentration. The relative productivities were calculated from the results obtained during the long-term continuous process as compared to the ones obtained at the beginning of the process/initial productivity.

### 2.10. High-Performance Liquid Chromatography (HPLC) Analysis of Fumaric Acid Concentration

Biotransformation products were analyzed by an HPLC (Shimadzu, Tokyo, Japan) equipped with a Gemini-NX reverse phase C18 column (Phenomenex, Torrance, CA, USA) and ultraviolet/visible (UV/Vis) detector. The separation was performed at room temperature with 0.1 M phosphoric acid aqueous solution (pH 2.9) as a mobile phase. At a flow rate of 0.5 mL/min, the residence time of fumaric acid, detected at 226 nm, was 2.4 min.

## 3. Results

### 3.1. Characterization of SA-PVA Copolymer Hydrogel

In order to develop an effective microreactor system between plates and with a biocatalyst immobilized in the hydrogel layer attached on the wall, it was of great importance to obtain as thin as possible film of a hydrogel. This would prevent diffusional limitations and enable efficient contact between the substrate molecules and the permeabilized yeast cells in the hydrogel. The thickness of the hydrogel layer was decreasing with increasing PVA concentration in the copolymeric hydrogel, with 200 μm being the thinnest layer obtained. Furthermore, the prepared hydrogel should be stable under operational conditions and would need to have a low swelling ratio because of a small reactor volume.

**Figure 2:**
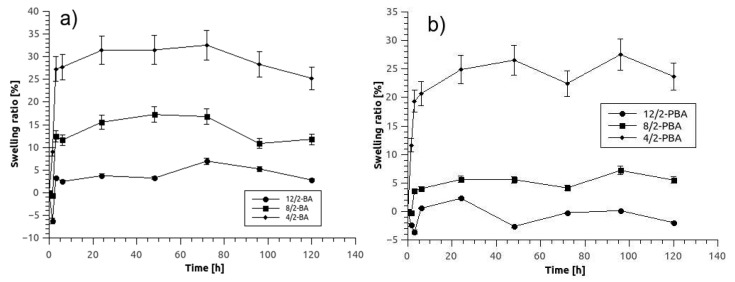
Swelling ratios of hydrogel samples of different compositions stated in legends (according to description in Table 1 crosslinked with (**a**) boric acid (BA) and (**b**) phenylboronic acid (PBA) as a function of incubation time in a demineralized water. Error bars indicate standard deviations of at least 3 replicates.

The results of the swelling behavior of the hydrogel samples are shown in Figure 2. It is evident that by raising the PVA concentration in the copolymer solution from 4% to 12% (*w*/*v*), the swelling ratio decreases from 25% to 3% after 120 h incubation time. This might be a consequence of increased PVA-borate crosslinking at higher PVA concentrations. The mechanism of PVA–borate crosslinking is believed to include a didiol complex, in which two diol units of PVA chain with one borate ion to form a crosslinked hydrogel [19]. Also, the swelling rate is smaller in samples that were crosslinked with PBA instead of BA which indicates that hydrogel samples crosslinked with PBA and with higher PVA concentrations would be favorable for microreactor application.

**Figure 3:**
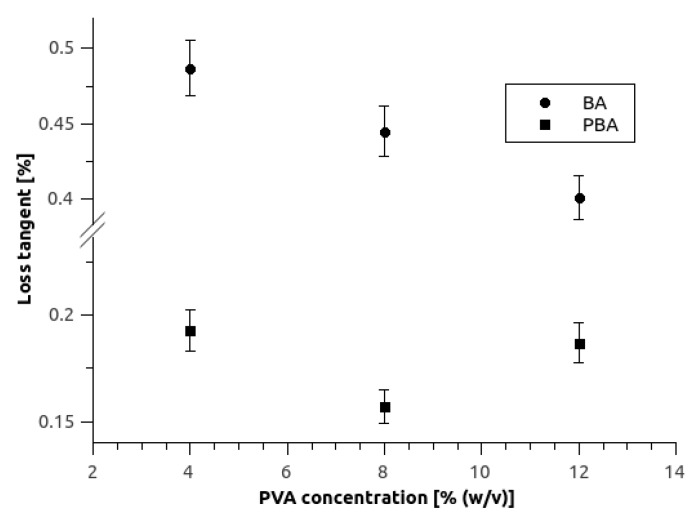
Average loss tangents of sodium alginate-polyvinyl alcohol (SA-PVA) hydrogel blends with 2% of SA, crosslinked with BA and PBA in relation to PVA concentration. Error bars indicate standard deviations of at least 3 replicates.

Rheological measurements revealed that all tested SA-PVA samples had average loss tangent parameter tan δ considerably lower than 1. This implies more solid-like structure since it has a bigger storage to loss moduli ratio [33]. From Figure 3 it is clear that BA hydrogel samples have bigger loss tangent value which indicates weaker and softer gel. On the other hand, with increasing PVA concentration, the tan δ value is decreasing, which indicates the creation of stronger gels. This is not the case with PBA samples however, where 12/2-PBA hydrogel is weaker than 8/2-PBA, as evident also from their handling. Namely, the 12/2-PBA hydrogel was more brittle, and it could break more easily. Since 8/2-PBA hydrogel displayed not only the best mechanical properties, but also good swelling ratio, it was selected for further use in the biotransformation studies.

### 3.2. Determination of the Immobilization Effectiveness Factor

**Figure 4:**
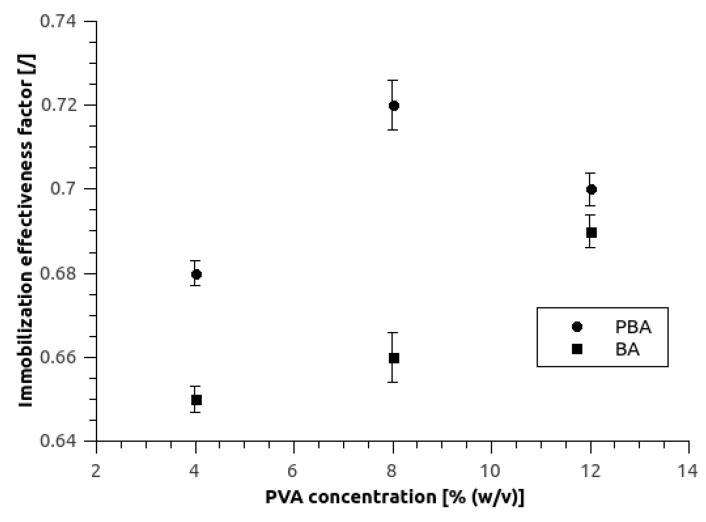
Immobilization effectiveness factor of BA and PBA hydrogel samples with 2% of SA in relation to PVA concentration. All reactions were carried at 5 mM substrate concentration, at 30 °C and at 150 rpm mixing speed. Error bars indicate standard deviations of at least 3 replicates.

Each immobilization technique affects the biocatalyst activity in some way, either with enzyme deformation or in our case by increased mass transfer of substrate to biocatalyst, which consequently affects the reaction rate. From the series of batch processes with free and immobilized enzyme and by using Equation (3), the effect of immobilization on biocatalyst activity was calculated for hydrogels of various compositions (Figure 4). In all developed hydrogels, the immobilization effectiveness factor (*η*) was above 0.65, which is more than satisfying as compared to the literature [34]. PBA hydrogels with immobilized yeast cells had higher *η* which is probably due to toxicity of boric acid toward *S. cerevisiae* cells that in turn lowered their catalytic activity [19]. The effectiveness factors varied from 0.65 to 0.72 for 4/2-BA and 8/2-PBA, respectively.

### 3.3. Biotransformation within a Microbioreactor

Conversion of fumaric to L-malic acid was further studied in a microbioreactor using one or two layers of hydrogel between two plates (Figure 1). A microbioreactor with two layers of cell-loaded hydrogel enabled to achieve a biocatalyst load of 188.5 (±4.5) mg_dw of cells_/mL, which is much higher when compared with the tubular microreactor containing surface-immobilized *S. cerevisiae* cells with the load of only 5.2 mg_dw of cells_/mL [6].

**Figure 5:**
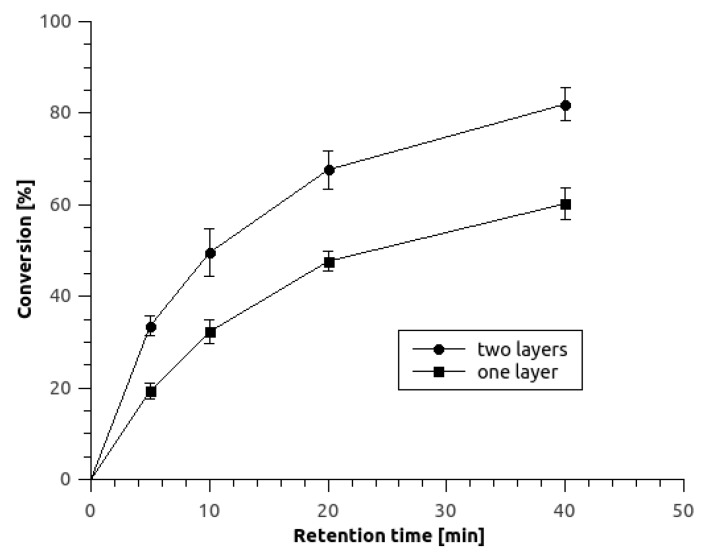
Comparison of fumaric acid conversion as a function of residence time within the microreactors using immobilized cells in one and two 8/2-PBA hydrogel layers at 30 °C and with 5 mM inlet substrate concentration at pH 7. Mean values of experimental data are presented by points with indicated standard deviations of at least 3 replicates.

As anticipated, two layers of hydrogel offering twice as much of the immobilized biocatalyst than a single hydrogel layer significantly improved the conversion at the outlet of the microbioreactor, as summarized in Figure 5. The highest outlet conversion obtained at 40 min residence time increased from 60% to 82% when using one or two-layered microreactor, respectively, providing the same process conditions. It shows that the conversion increase is not linear with the biocatalyst load, which indicates deeper substrate diffusion within a single hydrogel layer. Space time yield (volumetric productivity) obtained with 5 mM inlet substrate concentration and at retention time of 10 min for a double-layered microbioreactor was calculated to be 2.86 g/(L·h) (512 mM/day). At the same residence time and inlet substrate concentration, a single-layered microbioreactor achieved space time yield (STY) or volumetric productivity of 1.71 g/(L·h) (305 mM/day). Both results are lower than in the case of fumaric acid biotransformation using surface-immobilized permeabilized *S. cerevisiae* cells in a tubular microreactor of 24.5 µL volume with volumetric productivity of 616 mM/day [6], but substantially higher than that obtained in a membrane bioreactor of 1 dm^3^ volume with other strain of *S. cerevisiae* cells, where 174 mM/day was reported [28].

When comparing biocatalyst productivity, a 350 μL-volume microbioreactor with two hydrogel layers resulted in 2.08 mmol/g_ww of cells_/day. The comparison with other reactors and immobilization techniques used for the same biotransformation revealed that the above stated 24.5 µL-volume tubular microbioreactor and a 1 dm^3^-volume membrane bioreactor with *S. cerevisiae* cells, where biocatalyst productivities of 11.8 and 3.49 mmol/g_ww of cells_/day were achieved, respectively [6,28], could more efficiently make good use of the yeast cells. This indicates lower accessibility of the biocatalyst in deeper layers of the hydrogel in the microbioreactor between two plates, confirmed also with the calculation of the portion of active yeast cells inside the hydrogel layers revealing that only 8.5% of cells were active. Since the hydrogel layer is 200 μm thick and the average diameter of the *S. cerevisiae* cell is 2–15 μm [35], the solution lies in achieving thinner layers of the hydrogel enabling the exploitation of all immobilized cells. Further optimization of biocatalyst load and hydrogel layer thickness is, therefore, envisaged. On the other hand, the immobilization procedure used in this work is much more user-friendly, cheaper and less time consuming than the one yielding surface-immobilized cells as a biocatalyst. Also, we intend to further increase the STY and productivity (capacity) using also model-based design of the reactor/reaction set-up.

**Figure 6:**
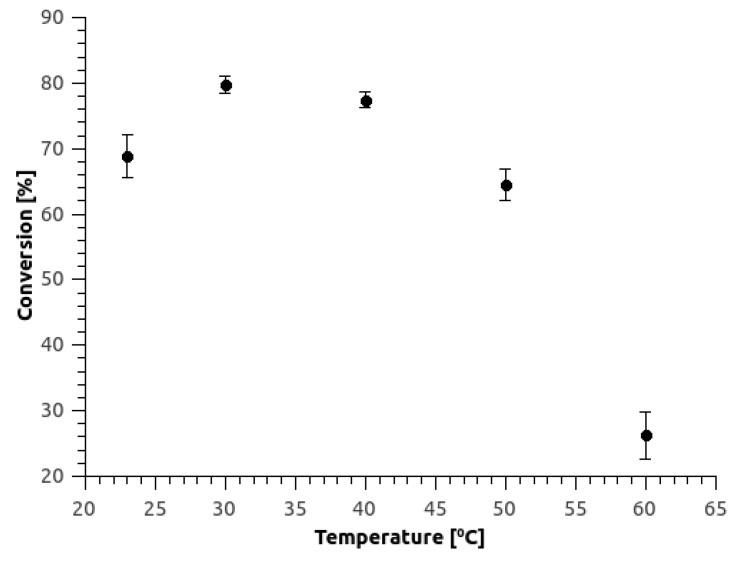
The influence of temperature on fumaric acid conversion. Inlet substrate concentration was 5 mM, while residence time was set at 30 min. The microreactor consisted of two 8/2-PBA hydrogel layers with a working volume of 350 (±5) μL and yeast cells concentration of 188.5 (±4.5) mg_dw of cells_/mL. Error bars indicate standard deviations of at least 3 replicates.

A microreactor with permeabilized *S. cerevisiae* cells in 8/2-PBA hydrogel in the layers on both sides of the plates was further used to study the temperature effect on the selected biotransformation process. Hydration of fumaric acid using fumarase in yeast cells is well-investigated reaction, but optimal temperature might shift due to the immobilization technique applied [19]. For permeabilized *S. cerevisiae* MZKI K86 in the free form the optimal temperature was found to be 30 °C [6]. From Figure 6 it is evident that the same cells immobilized in the copolymeric hydrogel layer performed at 30 °C, but were also very efficient at 40 °C. By contrast, the activity decreased by more than 10% at room temperature and at 50 °C, while further temperature increase resulted in substantial activity loss. With this we have confirmed that developed microbioreactor enabled very efficient in-operando process parameters evaluation in a short time with low material consumption, which is extremely beneficial as compared to conventional systems [3,7].

### 3.4. Determination of System Stability

**Figure 7:**
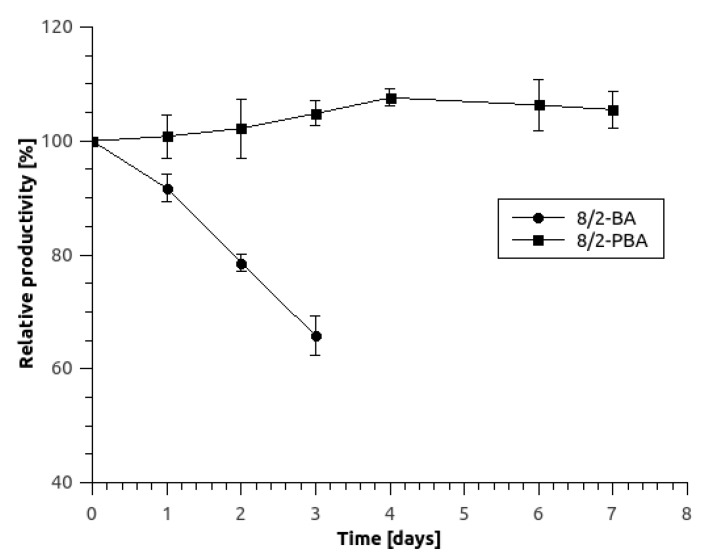
Relative productivity as a function of continuous-operation process time at room temperature. For the estimation of productivity, the flow rate of 5 μL/min was used and the inlet substrate concentration was 5 mM. Error bars indicate standard deviations of at least 3 replicates.

To evaluate the operational stability of the system, the microreactor was tested over a period of 7 days by continuous operation at 5 μL/min flow rate of the 5 mM fumaric acid. A microreactor with immobilized permeabilized yeast cells in a 8/2-PBA hydrogel with 200 μm thickness was assembled as previously described. As evident from Figure 7, the relative productivity of the microreactor with immobilized yeast cells in 8/2-PBA hydrogel was constant and no changes in physical or mechanical characteristics of the hydrogel were observed. In contrast, the relative productivity of 8/2-BA hydrogel with permeabilized *S. cerevisiae* cells decreased to 65% after 3 days while also showing fractures and cracks and final breaking after 4 days of continuous operation. The deactivation constant for 8/2-BA hydrogel productivity was calculated to be *k*_D_ = −0.475 h^−1^ and the half-life (*t*_1/2_) was 107.6 h. The stability was therefore significantly improved as compared to surface-immobilized yeast cell in a 24.5-μL tubular microbioreactor where relative productivity dropped to 10% after 4 days [6].

## 4. Conclusions

The immobilization of *S. cerevisiae* cells through entrapment in a porous structure of copolymeric hydrogel was demonstrated to be very efficient. Physico-chemical characterization of the hydrogels of various compositions prepared by different crosslinking agents revealed the best results for the hydrogel composed of 2% of SA and 8% of PVA, crosslinked with PBA. Furthermore, the retained activity was higher using PBA as a crosslinking agent. It was possible to integrate two layers of selected hydrogel with immobilized permeabilized *S. cerevisiae* cells in a microbioreactor between two plates enabling very high biocatalyst load. The results of continuous fumaric acid biotransformation in this system revealed high volumetric productivities and heavily improved stability as compared to other flow systems for L-malic acid production. Considering the ease of the immobilized biocatalyst preparation and stability, such hydrogel structures used within microreactors present huge potential for implementation in biocatalytic processes.

## Figures and Tables

**Table 1:** Composition of the tested hydrogels.

Hydrogel Name	Polyvinyl Alcohol (PVA) Concentration [% (*w*/*v*)]	Sodium Alginate (SA) Concentration [% (*w*/*v*)]	Crosslinker
4/2-BA	4	2	Boric acid
8/2-BA	8	2	Boric acid
12/2-BA	12	2	Boric acid
4/2-PBA	4	2	Phenylboronic acid
8/2-PBA	8	2	Phenylboronic acid
12/2-PBA	12	2	Phenylboronic acid
